# Evaluating Biomechanical and Viscoelastic Properties of Masticatory Muscles in Temporomandibular Disorders: A Patient-Centric Approach Using MyotonPRO Measurements

**DOI:** 10.3390/bioengineering12020097

**Published:** 2025-01-22

**Authors:** Daniele Della Posta, Ferdinando Paternostro, Nicola Costa, Jacopo J. V. Branca, Giulia Guarnieri, Annamaria Morelli, Alessandra Pacini, Gaetano Campi

**Affiliations:** 1 The Anatomical Network APS, Via Fermo 2c, 00182 Rome, Italy; osteodan@gmail.com (D.D.P.); nicolacosta.osteopatia@gmail.com (N.C.); 2 Department of Experimental and Clinical Medicine, Anatomy and Histology Section, University of Florence, 50134 Florence, Italy; ferdinando.paternostro@unifi.it (F.P.); giulia.guarnieri@unifi.it (G.G.); a.morelli@unifi.it (A.M.); alessandra.pacini@unifi.it (A.P.); 3 Institute of Crystallography, Consiglio Nazionale delle Ricerche (CNR), Via Salaria Km 29.300, 00015 Monterotondo, Italy; gaetano.campi@cnr.it

**Keywords:** temporomandibular joint, temporomandibular joint disorder, masticatory system, myotonic measurements, viscoelastic profile, anatomical network, osteo-myofascial network, physical statistics

## Abstract

The temporomandibular joint (TMJ) is essential for chewing and speaking functions, as well as for making facial expressions. However, this joint can be affected by disorders, known as temporomandibular disorders (TMDs), induced by complex causes that lead to limitations in daily activities. Building on the methodology and findings from our previous study on TMJ function, our research aims to apply the established criteria and norms to patients with TMDs. The primary goal is to evaluate the applicability and clinical relevance of these reference norms in predicting the severity and progression of TMJ disorders within a clinical population. Using non-invasive myotonic measurements, we evaluated 157 subjects, including both non-TMD-affected and TMD-affected individuals. To achieve optimal results, five primary parameters (frequency, stiffness, decrement, relaxation time, and creep) were analyzed using statistical–physical tools, providing quantitative *functionality degrees* across different previously examined clinical groups. The obtained results identified significant quantitative markers for early diagnosis and personalized treatment of TMJ disorders. This interdisciplinary approach leads to a deeper understanding of TMJ dysfunctions and makes a meaningful contribution to clinical practice, providing more precise tools for managing and treating this complex condition.

## 1. Introduction

The temporomandibular joint (TMJ) has a pivotal role in the masticatory system, enabling essential function such as chewing, speaking, and facial expressions [1]. Disorders affecting this joint, known as temporomandibular disorders (TMDs), encompass a range of clinical conditions that impact the TMJ, the muscles involved in mastication, or both, leading to significant discomfort, pain, and functional limitations for those affected [2]. The etiology of TMDs is complex and multifactorial, involving interactions between anatomical, physiological, psychological, and environmental factors, making diagnosis and treatment a challenging endeavor [3,4]. Malocclusions and craniofacial asymmetries may contribute to TMDs because variability or abnormalities occurring at the level of the joint capsule, the articular disc, or surrounding ligaments of the TMJ, for example, often underlie TMDs. These can reflect functional disruptions in the masticatory system; such disruptions include irregular jaw movement patterns, altered muscle tone, or bite force imbalances. Stress, anxiety, depression, and other psychosocial factors are also frequently associated with TMDs. Bruxism, a condition often exacerbated by stress, is a notable contributor. External influences such as trauma, prolonged dental procedures, or habits like gum chewing can trigger or exacerbate TMDs, together with other comorbidities such as autoimmune diseases, hormonal imbalances, and genetic predispositions play a significant role in susceptibility to and the progression of the disorder. Thus, the complexity and the widespread nature of TMDs globally, along with their impact on quality of life, highlights the need for improving diagnostic tools and therapeutic strategies [5,6,7]. Traditional diagnostic criteria have primarily relied mainly on clinical assessments and self-reported symptoms from patients. However, there is a notable absence of universally adopted, objective, quantitative measures to evaluate the functional state of the masticatory system, despite recent advancements in this area [8,9,10]. This gap has often resulted in variability in diagnosis and treatment outcomes, highlighting the need for standardized evaluation protocols that may provide a more comprehensive understanding of the biomechanical and viscoelastic properties of these disorders.

Our previous study introduced a novel method for assessing TMJ function by using non-invasive myotonic measurements. This approach established reference norms for the biomechanical and viscoelastic properties of the masticatory system in a healthy population [11]. Through statistical and physical analysis, we created a framework that quantifies the functional state of the TMJ and its components. This framework has potential applications for the early diagnosis and personalized treatment of TMDs. Building on this foundation, the current study applies these established norms to a group of patients diagnosed with TMDs based on internationally recognized criteria and who exhibit symptoms associated with the condition. Our goal is to validate the predictive value and clinical relevance of these reference norms in a pathological context, providing insights into the relationship between quantitative measures and the severity and progression of TMDs. By concentrating on patients with a confirmed diagnosis of TMD, this study aims to clarify the usefulness of biomechanical and viscoelastic profiling in enhancing diagnostic accuracy and tailoring therapeutic interventions for individuals suffering from TMDs. This study specifically focuses on masticatory muscles, analyzing their biomechanical and viscoelastic properties in relation to TMDs. This emphasis is crucial for understanding the contribution of muscle dysfunctions to the development and progression of TMD [12]. In summary, this study aims to evaluate how well-established reference norms for biomechanical and viscoelastic properties can predict the severity and progression of TMDs in a clinical setting. Specifically, it seeks to validate the effectiveness of non-invasive myotonic measurements in assessing TMD severity and improving diagnostic accuracy. Additionally, the study aims to identify key quantitative markers that can guide personalized treatment strategies, ultimately enhancing patient care and outcomes for individuals with TMDs. Ultimately, this research seeks to improve patient care and outcomes.

## 2. Materials and Methods

### 2.1. Subject Recruitment

For the present clinical study, aiming to contribute to clinical advances in the diagnosis and treatment of TMJ dysfunction, a total of 157 subjects were recruited and evaluated during visits to Dr. Daniele Della Posta’s Clinic. All the participants, both male and female, aged between 19 and 87 years old, were divided into subjects without TMDs and patients with TMDs; a specially structured questionnaire was used to assess the symptoms and impact of TMD in Axis II according to the diagnostic criteria (DC)/TMD [10]. All the subjects recruited for the study were excluded if they had severe neurological conditions, medical conditions significantly impairing masticatory function, or a history of major surgical interventions involving the TMJ.

### 2.2. Questionnaire

The questionnaire was administered to patients during their initial visits to Dr. Daniele Della Posta’s office. Each patient completed the questionnaire (as reported in Appendix A) in the presence of a healthcare professional who provided any necessary clarifications to ensure the proper understanding of the questions. The questionnaire comprises ten questions covering various aspects relevant to the assessment of temporomandibular disorders, including the frequency and intensity of pain, the impact of pain on daily life, sleep quality, oral functionality, psychological symptoms, and the frequency of teeth clenching/grinding.

### 2.3. Data Collection

The data collected through the questionnaire were analyzed using standard statistical methods to assess the prevalence and severity of TMD symptoms in relation to various considered aspects. Responses were coded and entered into an electronic database, allowing for the easy management and analysis of the data. The categorized responses were used to identify levels of pain, impact, and functional and psychological difficulties, to classify patients according to the DC/TMD diagnostic criteria [10]. This methodological approach allowed for a detailed and accurate assessment of the conditions of patients with TMDs, facilitating the identification of the patients’ clinical conditions.

### 2.4. Analysis

Based on the criteria used in a previous study [11], the data obtained from the MyotonPRO (MYOTON AS, Tallin, Estonia) measurements of the masticatory muscles were subsequently processed with updated software for correlation analyses [13]. The MyotonPRO device is designed to quantify the tone, biomechanical, and viscoelastic responses of muscles [14,15,16], evaluating the following specific biomechanical parameters of the masticatory muscles:

1. **Frequency** (F): This parameter reflects the rate of muscle contractions, providing insights into muscle function and responsiveness.
2. **Stiffness** (S): Stiffness indicates the resistance of the muscles to deformation, which can be crucial in understanding muscle tone and health.
3. **Decrement** (D): This measures the decline in muscle response over time, which can help assess muscle fatigue and endurance.
4. **Mechanical Stress Relaxation Time** (R): This parameter assesses how quickly a muscle relaxes after being contracted, providing information about muscle recovery dynamics.
5. **Creep** (C): Creep evaluates the deformation of muscles under sustained load, which can indicate how muscles adapt to prolonged stress or tension.

This is achieved by applying a brief mechanical impulse (lasting 15 ms and exerting a force of 0.58 N) to the skin surface above the muscle [17]. The device’s testing end, held perpendicular to the skin, induces a mechanical deformation in the underlying tissue. An integrated 3-axis digital acceleration sensor captures the dynamic response of the muscle, producing an accelerogram characterized by damped oscillations. The recorded signal is analyzed in real time by embedded software (Myoton Desktop Software v.5.0.0.232), which extracts five key parameters, A*^m^*: frequency (F where *m* = 1), stiffness (S, where *m* = 2), decrement (D, where *m* = 3), mechanical stress relaxation time (R, where *m* = 4), and creep (C, where *m* = 5). Measurements taken by the MyotonPRO at the bone insertion points of the masticatory muscles form osteon-muscular networks. These networks, consisting of 17 nodes, are schematized and described in detail in our previous articles [10,11,16,17,18]. The measurements are conducted at the contact points between two nodes that are situated on the 20 links (l = 1, …, 20). These links are grouped into four cycles (cy = I, II, III, IV), each closing on the same mandibular node.

**Figure 1:**
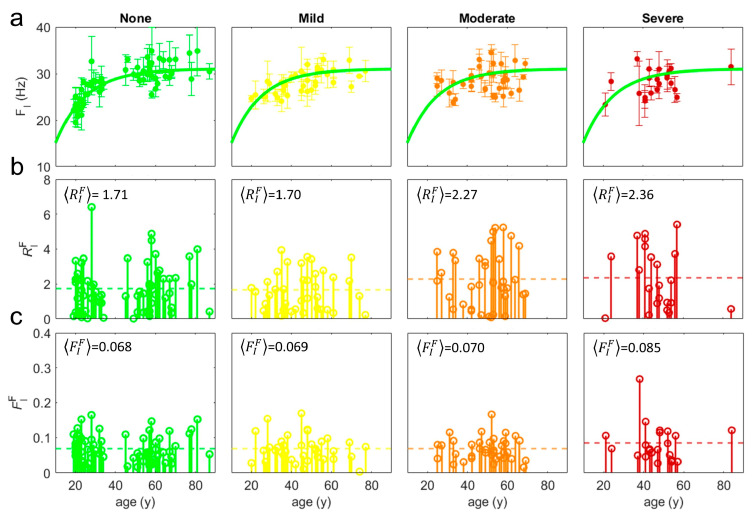
(**a**) Measured values of frequency (full circles) on the first cycle, $A_{cy}^{m}\left(y\right)$, with *A^m^* = F and *cy* = I, for patients categorized into four functional groups based on the severity of the dysfunction: mild (yellow), moderate (orange), severe (red), and no dysfunction (green). The thick green line represents the physiological trend line $T_{cy}^{A^{m}}\left(y\right)$ of frequency in I-cycle derived from healthy individuals [8]. This trend line was established through exponential fitting of frequency as a function of age, allowing for a comparative analysis of patient measurements against normative data. (**b**) Residuals ${\left[R_{cy}^{A^{m}}\right]}_{f}$ representing the absolute differences between measured values $A_{cy}^{m}$ and the corresponding trend line values $T_{cy}^{A^{m}}$, indicating that higher residuals correlate with increased dysfunction severity, thus reflecting lower functionality. (**c**) Fluctuations ${\left[F_{cy}^{A^{m}}\right]}_{f}$ calculated as the ratio of the standard deviation of measured values $\sigma (A_{cy}^{m}\left(y\right))$ to their average $\langle A_{cy}^{m}\left(y\right)\rangle$. An upward trend in fluctuations from no dysfunction to severe dysfunction suggests that increased variability in muscle responses may negatively impact functionality. The dashed lines in (**b**,**c**) are the average ${\langle R_{cy}^{A^{m}}\rangle }_{f}$ and ${\langle F_{cy}^{A^{m}}\rangle }_{f}$ of residuals and fluctuations data, respectively, in each functional group.

Due to the specific different values of *A^m^* measured in links belonging to the different cycles, *cy*, we have studied the evolution of our measurements, *A^m^,* averaged on each of the four cycles, $A_{cy}^{m}$*,* as a function of the patients’ age [11]. For physiological patients, without any diagnosed dysfunction, we have built physiological trend lines, $T_{cy}^{A^{m}}$, by fitting the exponential behavior of *A^m^* as a function of time age [11]. In Figure 1a, we show the $A_{cy}^{m}$ values (full circles) alongside the trendline $T_{cy}^{A^{m}}$ (green thick line) with *A^m=^*^1^ = F (Hz) and *cy* = I; these are provided for the four different groups formed from the total 157 patients by different diagnosis with “none”, “mild”, “moderate”, and “severe” dysfunctions. The different functionality groups are represented by different colors: green, yellow, orange, and red for no, mild, moderate, and severe dysfunctions.

We aimed to establish a functionality degree for each patient. To achieve this, we used the following quantities:

(i) The *residuals*, $R_{cy}^{A^{m}}$, as the distance between the measured $A_{cy}^{m}$ and the physiological value on the trendline $T_{cy}^{m}$ for each cycle, *cy* = I, II, III, IV, at a specific age, *y*:

(1) $$R_{cy}^{A^{m}}\left(y\right)=\left|A_{cy}^{m}\left(y\right)-T_{cy}^{A^{m}}\left(y\right)\right|$$

(ii) The *fluctuations*, $F_{cy}^{A^{m}}$, given by the ratio between the standard deviation of the measured $A_{cy}^{m}$, $\sigma (A_{cy}^{m})$ and its average $\langle A_{cy}^{m}\rangle$:

(2) $$F_{cy}^{A^{m}}\left(y\right)=\sigma (A_{cy}^{m}\left(y\right))/\langle A_{cy}^{m}\left(y\right)\rangle$$

The residual is a quite intuitive quantity giving the deviation of the patient’s measured $A_{cy}^{m}\left(y\right)$ from the expected $T_{cy}^{m}\left(y\right)$ (predicted value on the trendline) for their specific age, *y*. The smaller the residual, the closer the measured $A_{cy}^{m}$ is to the trendline, indicating a higher functionality. Conversely, a larger residual would indicate a lower functionality. The fluctuations represent the *variability* of $A_{cy}^{m}$ in the *m*-measurements on the *cy*-cycle. In living systems, this variability can be not random and can positively impact functionality. This phenomenon is known as *correlated disorder*. Conversely, in other cases, such variability can be detrimental, reducing functionality [19,20,21,22,23]. Thus, recognizing when variability enhances functionality versus when it impairs it can lead to more accurate diagnoses and tailored treatments. In Figure 1b,c, we show the *residuals,* $R_{I}^{F}$, and the *fluctuations,* $F_{I}^{F}$, of the frequency (*A*^1^ = F) calculated on the first cycle, *cy* = I, for each functional group, *f*, with *f* = mild, moderate, severe, or no dysfunction. The average of each set of ${\left[R_{I}^{F}\right]}_{f}$ and ${\left[F_{I}^{F}\right]}_{f}$ are represented by the dashed lines and provides us the average residuals, ${\langle R_{I}^{F}\rangle }_{f}$, and average fluctuations, ${\langle F_{I}^{F}\rangle }_{f}$. We can already observe here a tendency of these mean values to increase from the group (green) without dysfunctions to the group (red) with severe dysfunctions.

**Figure 2:**
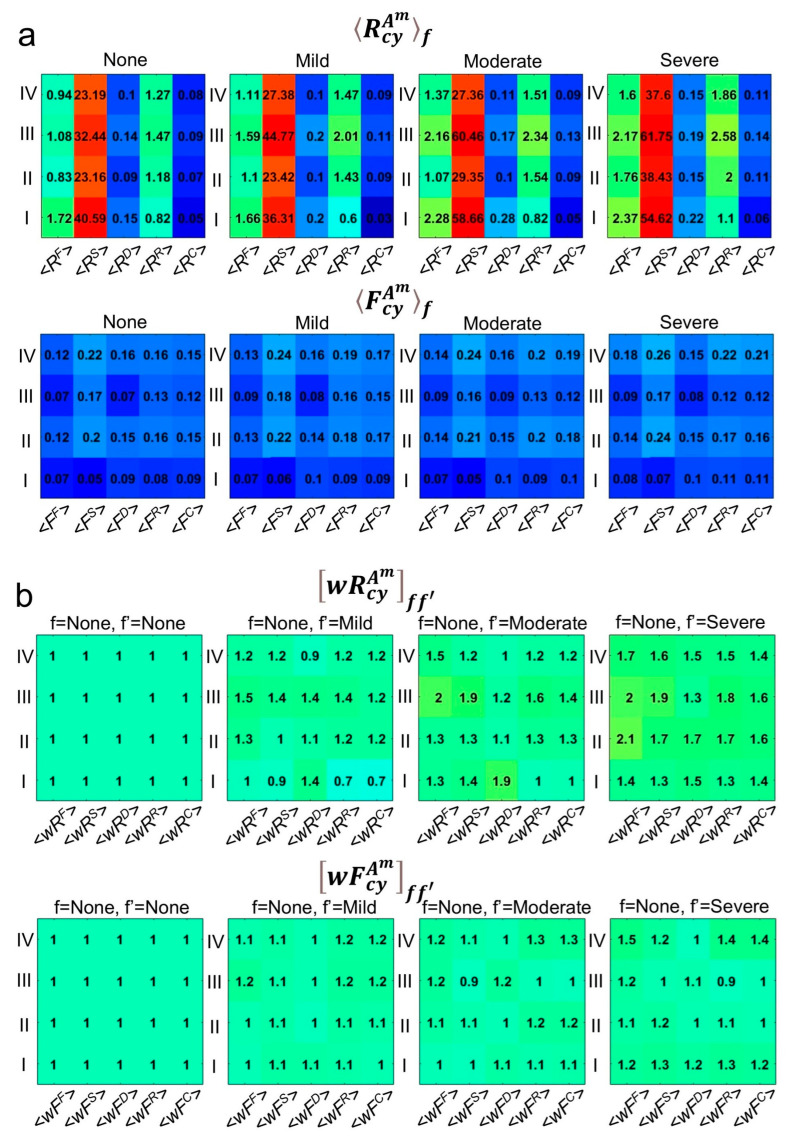
(**a**) Average residuals ${\langle R_{cy}^{A^{m}}\rangle }_{f}$ between the measured $A_{cy}^{m}$ and the trend line $T_{cy}^{A^{m}}$ and average fluctuations, ${\langle F_{cy}^{A^{m}}\rangle }_{f}$. *< >_f_* represents the averaging across different functional groups of patients with mild, moderate, severe and no dysfunction illustrating the deviation of measured values from expected norms. This analysis provides insight into how well each group aligns with physiological expectations, with larger averages indicating greater dysfunction. (**b**) Calibration matrices ${\left[{wR}_{cy}^{A^{m}}\right]}_{ff^{\prime }}$ and ${\left[{wF}_{cy}^{A^{m}}\right]}_{ff^{\prime }}$ represent the weights used in summing residuals and fluctuations, in Equations (3) and (4), respectively. These matrices quantify deviations of measurements in groups with mild, moderate, and severe dysfunction from those without dysfunction, facilitating a more nuanced understanding of how the dysfunction degree affects muscle properties. The “jet colormap” gradient (ranging from blue to red) was used in order to visually represents the numerical values in the matrices, highlighting variations and patterns in the data.

The averaged residuals and fluctuations calculated for *m* = F and *cy* = I in Figure 1, can be calculated for all cycles, *cy* = I, II, III, and IV and all measurements *m* = F, S, D, R, C, providing *m* × *cy* matrices, ${\langle R_{cy}^{A^{m}}\rangle }_{f}$ and ${\langle F_{cy}^{A^{m}}\rangle }_{f}$, where *f* is a single functional group (see Figure 2a). It appears clear the tendency of (*m*, *cy*) elements in ${\langle R_{cy}^{A^{m}}\rangle }_{f}$ and ${\langle F_{cy}^{A^{m}}\rangle }_{f}$ matrices to assume largest value going from patients with no dysfunction towards patient with diagnosed severe dysfunctions. This trend suggests that as the masticatory system becomes more dysfunctional, the residuals and fluctuations become more pronounced. This observation led us to establish, for each patient, with age y, *partial* functionality degrees, ${Rw}_{f}^{m}$ and ${Fw}_{f}^{m}$, for each measurement, *A^m^*, given by the summation of the calculated residuals and fluctuations on the different cycles, *cy*:

(3) $${Rw}_{f}^{m}\left(y\right)=\sum_{cy}{\left[{wR}_{cy}^{A^{m}}\right]}_{ff^{\prime }}*R_{cy}^{A^{m}}\left(y\right)$$

(4) $${Fw}_{f}^{m}\left(y\right)=\sum_{cy}{\left[{wF}_{cy}^{A^{m}}\right]}_{ff^{\prime }}*R_{cy}^{A^{m}}\left(y\right)$$

These sums are weighted by the ${\left[{wR}_{cy}^{A^{m}}\right]}_{ff^{\prime }}$ and ${\left[{wF}_{cy}^{A^{m}}\right]}_{ff^{\prime }}$ matrix coefficients

(5) wRcyAmff′=RcyAmfRcyAmf′

(6) wFcyAmff′=FcyAmfFcyAmf′

where *f’* is the group with no dysfunctions, while *f* represents the other groups with mild, moderate, and severe dysfunctions. The ${\left[{wR}_{cy}^{A^{m}}\right]}_{ff^{\prime }}$ and ${\left[{wF}_{cy}^{A^{m}}\right]}_{ff^{\prime }}$ are called *calibration matrices* and represent how much the measurements in groups with mild, moderate, and severe dysfunction deviate from the functional group. These matrices are reported in Figure 2b.

To transform the *residuals* ${Rw}_{f}^{m}\left(y\right)$ and *fluctuations* ${Rw}_{f}^{m}\left(y\right)$ in functionality indices varying on an increasing and expanded scale of values, between 0 and 100, we have first calculated the logarithm of ${Rw}_{f}^{m}\left(y\right)$ and ${Rw}_{f}^{m}\left(y\right)$:

(7) $$P_{R}^{m}\left(y\right)=ln\left({\left|{Rw}_{f}^{m}\left(y\right)\right|}^{-1}\right)$$

(8) $$P_{F}^{m}\left(y\right)=ln\left({\left|{Fw}_{f}^{m}\left(y\right)\right|}^{-1}\right)$$

and then we have normalized them in the [0–100] range

(9) $$P_{RN}^{m}\left(y\right)=100\times \frac{P_{R}^{m}\left(y\right)-min\left[P_{R}^{m}\left(y\right)\right]}{max\left[P_{R}^{m}\left(y\right)\right]-min\left[P_{R}^{m}\left(y\right)\right]}$$

(10) $$P_{FN}^{m}\left(y\right)=100\times \frac{P_{F}^{m}\left(y\right)-min\left[P_{F}^{m}\left(y\right)\right]}{max\left[P_{F}^{m}\left(y\right)\right]-min\left[P_{F}^{m}\left(y\right)\right]}$$

**Figure 3:**
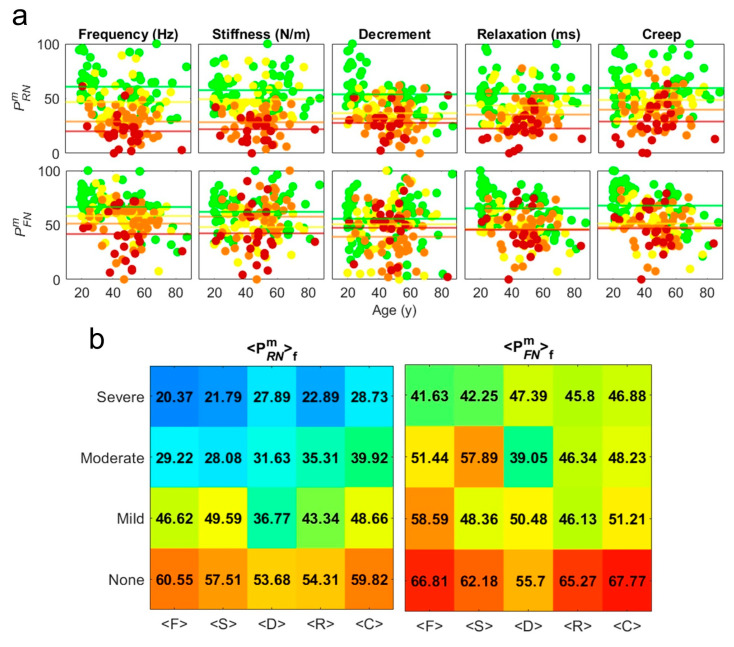
(**a**) Normalized partial functionality indices ${\left[P_{RN}^{m}\right]}_{f}$ and ${\left[P_{FN}^{m}\right]}_{f}$ scaled between 0 (largest dysfunctionality) and 100 (largest functionality) in each group of patients with different functionality, *f*, indicated by the different colors. These indices were derived from logarithmic transformations of residuals and fluctuations, followed by normalization across all patient groups. The continuous horizontal lines represent average normalized values ${\langle P_{RN}^{m}\rangle }_{f}$ and ${\langle P_{FN}^{m}\rangle }_{f}$, providing a clear visual representation of functional status across varying degrees of dysfunction severity. (**b**) Matrices containing numeric values of average normalized indices ${\langle P_{RN}^{m}\rangle }_{f}$ and ${\langle P_{FN}^{m}\rangle }_{f}$ for each patient group illustrate how functionality varies among individuals with differing levels of dysfunction. The “jet colormap” gradient (ranging from blue to red) was used in order to visually represents the numerical values in the matrices, highlighting variations and patterns in the data.

In this way, we have established the *partial functionality indices*, $P_{RN}^{m}$ and $P_{FN}^{m}$, shown in Figure 3a, on a user-friendly scale from 0 (*largest dysfunctionality*) to 100 (*largest functionality*).

The average of $P_{RN}^{m}$ and $P_{FN}^{m}$ datasets in each functional group, gives us ${\langle P_{RN}^{m}\rangle }_{f}$ and ${\langle P_{FN}^{m}\rangle }_{f}$, (continuous lines in Figure 3a) represented as matrices in Figure 3b. Here, the values of each column, *m*, are considered as *partial levels* of the *functionality scale*, allowing us to classify the type of functionality in a patient, for each measurement *A^m^*. It is important to note that the extent of this variability differs across each (m) measurement. This finding highlights the complex nature of living systems, where increased dysfunctionality is associated with greater variability, potentially reflecting underlying compensatory mechanisms or instability within the system [19,20,21,22,23].

Finally, we have been able to extract the total functionality indices, $P_{RT}$ and $P_{FT}$, integrating the partial indices $P_{RN}^{m}$ and $P_{RN}^{m}$. At this aim we have summed these partial indices for all the five *m*-measurements using ${\langle P_{RN}^{m}\rangle }_{f}$ and ${\langle P_{RN}^{m}\rangle }_{f}$ as weight matrices:

(11) $$P_{RT}\left(y\right)=\sum_{m,f}{\langle P_{RN}^{m}\rangle }_{f}*P_{RN}^{m}\left(y\right)$$

(12) $$P_{FT}\left(y\right)=\sum_{m,f}{\langle P_{RN}^{m}\rangle }_{f}*P_{RN}^{m}\left(y\right)$$

In analogy with the partial indices, we have expressed the total functionality indices in the [0–100] scale to obtain $P_{RNT}$ and $P_{FNT}$:

(13) $$P_{RNT}\left(y\right)=100*\frac{P_{RT}\left(y\right)-min\left[P_{RT}\left(y\right)\right]}{nax\left[P_{RT}\left(y\right)\right]-min\left[P_{RT}\left(y\right)\right]}$$

(14) $$P_{FNT}\left(y\right)=100*\frac{P_{FT}\left(y\right)-min\left[P_{FT}\left(y\right)\right]}{nax\left[P_{FT}\left(y\right)\right]-min\left[P_{FT}\left(y\right)\right]}$$

**Figure 4:**
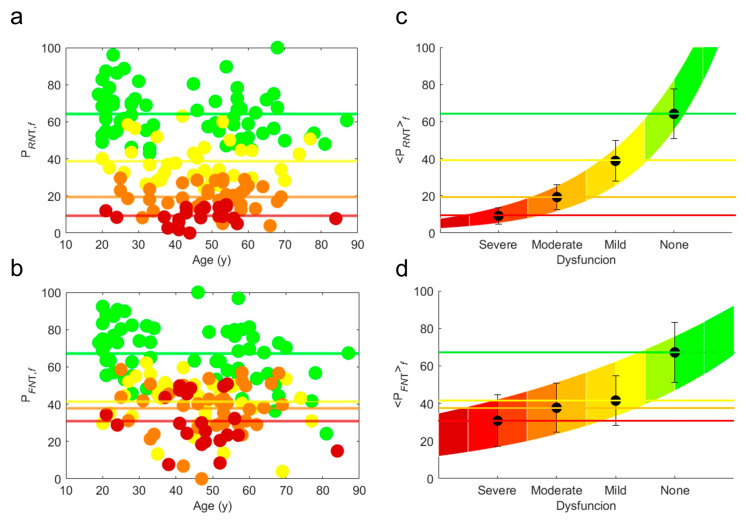
Total functionality indices (**a**) $P_{RNT}$ and (**b**) $P_{FNT}$, grouped by (yellow full circles) mild, (orange full circles) moderate, (red full circles) severe, or (green full circles) no dysfunction diagnosed for the 157 patients. The colored horizontal lines are the averages of all ages of patients belonging to the different functional groups. The average ${\langle P_{RNT}\rangle }_{f}$ and ${\langle P_{FNT}\rangle }_{f}$ are highlighted by black full circles with their error bar on a chromatic color scale in panels (**c**,**d**), respectively. The colored zone is comprised between two exponential lines given by Equations (15) and (16) covering the error bars across the four functional groups.

Figure 4a,b show P*_RNT_* and P*_FNT_* for the four diagnosed groups of patients. In the same figures, we show the average of P*_RNT_* and P*_FNT_* in each functional group, *f*, (${\langle P_{RNT}\rangle }_{f}$ and ${\langle P_{FNT}\rangle }_{f}),$ as horizontal lines whose color correspond with the different functional group. Figure 4c,d show ${\langle P_{RNT}\rangle }_{f}$ and ${\langle P_{FNT}\rangle }_{f}$ with their own standard deviation (error bars) as a function of *f*. We observe the clear trends of ${\langle P_{RNT}\rangle }_{f}$ and ${\langle P_{FNT}\rangle }_{f}$ increasing exponentially as:

(15) $${\langle P_{RNT}\rangle }_{f}=8.64e^{0.52f}$$

(16) $${\langle P_{FNT}\rangle }_{f}=15.52e^{0.33f}$$

Here, the values of ${\langle P_{RNT}\rangle }_{f}$ and ${\langle P_{FNT}\rangle }_{f}$ are considered as *levels* of the *total functionality scale*, allowing us to classify the type of functionality in a patient.

The color gradient has been built by using two colors per level from red (lower functionality) to green (high functionality), passing from yellow, thus imitating road signs.

To classify the measurements in a patient, we employed a pure correlation method that, without any clinical considerations, which assigns a functionality degree based on the correlation between the patient’s measurements and physiological lines drawn from a group of patients with no dysfunction. First, we constructed *m* physiological maps (*N_p,f_* × *L*), where *m* = 5 represents the number of measurements, $A_{L}^{m}\left(y\right)$. Here, *N_p,f_* denotes the number of patients with no dysfunction, and *L* = 20 is the number of links in the network [11]. Each patient measurement, *m*, consisting of *L* data points, is then correlated with the corresponding physiological maps for patients of the same age. It is expected that a more functional patient will have a higher correlation coefficient, $c_{m,f}(y)$. We emphasize that this method is purely numerical and does not consider any clinical diagnosis of the examined patient. By averaging the correlation coefficients obtained from each measurement, *m*, we have established a third functionality index.

(17) $$P_{cT}\left(y\right)={\langle c_{m,f}(y)\rangle }_{m}$$

**Figure 5:**
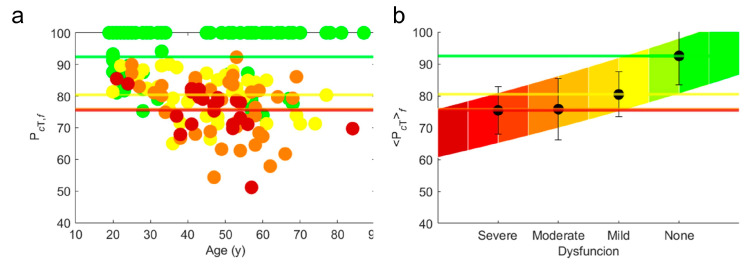
Total functionality indices (**a**) $P_{cT}$ grouped by (yellow full circles) mild, (orange full circles) moderate, (red full circles) severe, or (green full circles) no dysfunction diagnosed for the 157 patients. The colored horizontal lines are the averages of all the ages of patients belonging to the different functional groups. The average (**b**) ${\langle P_{cT}\rangle }_{f}$ with its error bar on a chromatic color scale. The colored zone is comprised between two exponential lines covering the error bars in the four functional groups.

Physiological maps measured on the 20 links in functional patients have been studied in [11]. In Figure 5, we show the total functionality indices. 

In summary, we have analyzed MyotonPRO measurements to evaluate the biomechanical and viscoelastic properties of masticatory muscles in patients with TMDs. Key parameters such as frequency, stiffness, decrement, relaxation time, and creep are extracted from dynamic responses to mechanical impulses. By comparing these measurements against established physiological trend lines, we assess functionality defining a quantitative scale and indices through residuals and fluctuations calculations. This is crucial for improving diagnostic accuracy and tailoring personalized treatment strategies for masticatory system dysfunction.

## 3. Results

The data obtained from MyotonPRO measurements of the masticatory muscles, categorized into four functional groups—no dysfunction, mild dysfunction, moderate dysfunction, and severe dysfunction—provides key insights into the biomechanical properties of these muscles. The five primary parameters measured—frequency, stiffness, decrement, relaxation time, and creep—were analyzed using advanced correlation methods to assess the functionality of the masticatory network. These analyses focused on identifying patterns and deviations from physiological norms that characterize different levels of dysfunction.

### 3.1. Biomechanical Properties by Functional Group

The four functional groups are detailed as follows:

**I. No Dysfunction**: Patients in this group showed consistent values close to the physiological norms for all parameters. For example, stiffness values remained within the normal range (300–750 N/m), and relaxation times were shorter, indicating healthy muscle function. The residuals and fluctuations were minimal, suggesting high alignment with the physiological trend lines established for healthy individuals. Additionally, these measurements confirmed the stability and functionality expected in patients without masticatory system impairments.

**II. Mild Dysfunction**: This group exhibited slight deviations in biomechanical properties, particularly in terms of relaxation time and stiffness. The average residuals for frequency and stiffness increased slightly compared to the no dysfunction group, indicating the onset of muscle tension or reduced elasticity. However, these deviations remained moderate, with fluctuations still relatively controlled, reflecting a mild level of muscle impairment. The data suggest that these patients are in the early stages of biomechanical alteration, where compensatory mechanisms might still be effective.

**III. Moderate Dysfunction**: Patients in this category displayed significant deviations from the physiological norms. Stiffness values often exceeded 800 N/m, and relaxation times became prolonged, sometimes doubling the values seen in healthy individuals. Decrement and creep values also reflected decreased energy dissipation ability and altered muscle behavior under prolonged stress. The residuals and fluctuations for these parameters showed notable increases, confirming the reduced functionality of the masticatory muscles. These changes highlight a transitional phase where the system’s ability to compensate is progressively diminishing.

**IV. Severe Dysfunction**: In this group, the biomechanical properties were the most significantly altered. Stiffness values soared beyond 900 N/m, indicating highly tense and rigid muscles. Relaxation times were substantially extended, with some patients showing values three times higher than those of the no dysfunction group. Decrement and creep metrics also pointed to severely impaired muscle function. The residuals and fluctuations reached their highest levels, emphasizing the profound divergence from the healthy trend lines. These results clearly illustrated the advanced level of dysfunction within the masticatory system for these patients. 

### 3.2. Functional Indices Derived from Residuals and Fluctuations

To further evaluate the functionality of the masticatory system, indices were derived from the residuals, $P_{cy}^{A^{m}}$, and fluctuations, $\varphi_{cy}^{A^{m}}$, calculated for each group. These indices reflect how closely the measured parameters align with physiological trends (residuals) and the variability within the parameters (fluctuations).

Summarized findings:

- Residuals: Residuals quantify the deviation of measured values from expected physiological norms. Larger residuals indicate reduced alignment and lower functionality. For instance, patients in the severe dysfunction group consistently showed the highest residual values, reflecting significant impairment.
- Fluctuations: Fluctuations represent the variability of measurements within a group. While some variability is indicative of natural adaptability, excessive fluctuations—as observed in the severe dysfunction group—suggest instability and diminished functional capacity.

The average residuals, $P_{cy}^{A^{m}},$ and fluctuations, $\varphi_{cy}^{A^{m}}$, were computed across the four functional groups, highlighting a clear trend of increasing dysfunction. These trends are visualized in Figure 1 (panels b and c), where the data are plotted against physiological trend lines values $T_{cy}^{A^{m}}$ (Figure 1a).

Functional classification and implications:

By analyzing the residuals and fluctuations across different parameters, it was possible to establish a functionality scale for each patient. This scale ranges from 0 (severe dysfunction) to 100 (optimal functionality), allowing for a nuanced classification of functional states. These indices provide valuable insights for clinical assessments and underscore the potential of quantitative tools in improving diagnostic precision.

However, there are instances where the clinical diagnosis based on questionnaires does not correspond to the instrumental measurements. This discrepancy highlights the importance of combining quantitative and qualitative assessments to capture a more comprehensive picture of masticatory system functionality. These findings emphasize the need for integrated diagnostic approaches to address latent dysfunctions effectively.

## 4. Discussion

The biomechanical data collected from MyotonPRO measurements provide valuable clinical insights for assessing and managing muscle function, including the masticatory muscles and their associated dysfunctions [24,25,26]. By analyzing specific key parameters—such as frequency, stiffness, decrement, relaxation time, and creep—across varying levels of dysfunction severity, we can achieve a better understanding of how these muscles respond under different pathological conditions [27,28]. Additionally, the correlation between these measurements and patients’ clinical presentations has significant implications for diagnosis, treatment, and prognosis. The biomechanical and viscoelastic properties of masticatory muscles analyzed in this study are key parameters for understanding the pathophysiology of TMDs. Muscle stiffness, decrement, and relaxation time, measured using the MyotonPRO device, are directly correlated with muscle functionality and the interaction between muscles and temporomandibular joints [29,30].

The collected data show that alterations in these biomechanical properties are associated with joint dysfunctions, suggesting that muscle dysfunctions not only reflect altered local biomechanics but may also affect joint movement and the stability of the masticatory system. These interactions, often overlooked in traditional literature, highlight the need for quantitative diagnostic approaches.

Moreover, our study adopts an interdisciplinary approach, integrating biomechanics principles to bridge the gap between traditional qualitative assessments and objective metrics. For instance, results related to muscle stiffness and decrement provide a direct indication of the degree of muscular adaptation to joint dysfunctions. These findings are supported by recent biomechanical studies [12,31,32]. Additionally, the compensatory muscle activation observed under load conditions underscores the importance of further exploring these biomechanical aspects even in static studies.

The data from patients classified as having no dysfunction or only mild dysfunction highlight values closely aligned with physiological norms, emphasizing the importance of early intervention for subtle deviations. Similar results have been observed in several studies, which underscore the utility of tools like MyotonPRO in identifying early-stage muscle dysfunctions [13,14]. Notably, the finding that patients with mild dysfunction showed muscle stiffness values comparable to those without dysfunction underscores the possibility that clinical classifications based solely on symptoms may overlook underlying biomechanical changes. This observation emphasizes the need to incorporate objective measurements, such as those provided by MyotonPRO, into routine diagnostic processes. This is especially important for the early detection of dysfunctions that may not yet manifest overt symptoms.

Conversely, patients with moderate–severe dysfunction displayed significant deviations across all measured parameters, including elevated stiffness and prolonged relaxation times, consistent with other studies, highlighting the role of biomechanical disruptions in advanced TMD cases [15]. These objective measures provide clear and quantifiable evidence of dysfunction that correlates with the patients’ clinical conditions. Identifying specific parameters that deviate from normal physiological values allows for a more accurate understanding of the functional state of the masticatory muscles, aiding in the classification of the severity of TMD.

The differences in biomechanical properties across the dysfunction spectrum indicate that therapeutic approaches should be customized based on the specific biomechanical deficits identified in each patient. For example, patients with mild dysfunction, characterized by moderate stiffness and slightly prolonged relaxation times, may benefit from conservative interventions such as physiotherapy. These interventions could focus on improving muscle elasticity and reducing tension through techniques like myofascial release, stretching, and progressive relaxation exercises.

In contrast, for patients with moderate–severe dysfunction, the marked increase in muscle stiffness, extended relaxation times, and abnormal decrement values underscore significant impairments in masticatory muscle function, consistent with findings that associate these alterations with chronic pain conditions, thus highlighting the need for more intensive and potentially multidisciplinary treatment strategies [33]. The increased stiffness and poor energy dissipation—evident through decrement and creep values—suggest that osteopathic intervention may be necessary to rebalance muscle tension and improve overall musculoskeletal alignment. These therapeutic options could be combined with dental approaches, such as occlusal adjustments or splint therapy, to effectively address both the muscular and skeletal components of TMD.

Moreover, the clear differences in functionality indices among various dysfunction groups highlight the potential of using these indices as a tool to monitor treatment progress. By tracking changes in parameters such as stiffness and relaxation time over time, clinicians can evaluate the effectiveness of therapeutic interventions and make necessary adjustments. This objective tracking offers a more reliable measure of patient improvement than relying solely on symptom-based reports.

The progression from mild to severe dysfunction, characterized by increasing residuals and fluctuations in biomechanical parameters, underscores the importance of early intervention. Patients in the mild dysfunction group, who exhibit only slight deviations from physiological norms, can benefit significantly from early, targeted therapies to prevent further deterioration. In contrast, patients with severe dysfunction, showing marked deviations across all measured parameters, may undergo a more prolonged and complex recovery process. The noticeable decline in functionality among these patients emphasizes the risk of further muscle deterioration if timely and appropriate treatment is not initiated.

Additionally, the correlation between biomechanical measures and clinical severity allows for more accurate prognostic predictions. For example, patients displaying higher stiffness and prolonged relaxation times may be at an increased risk of chronic pain and functional limitations if their condition is not effectively managed [34,35]. Thus, the validation of devices like MyotonPRO highlights their reliability in assessing biomechanical properties and advancing clinical diagnosis [36]. Specifically for TMDs, the data obtained by MyotonPRO aid in identifying individuals at risk, allowing clinicians to implement more proactive and timely interventions, thereby reducing the long-term impact of TMD.

The data strongly advocate for a multidisciplinary approach to managing masticatory muscle dysfunctions. MyotonPRO’s ability to quantify various biomechanical parameters, which are often not captured by traditional clinical evaluations, facilitates the integration of diverse therapeutic modalities [36]. Thus, a clear distinction in biomechanical parameters among the severity groups emphasizes the need for personalized treatment plans. Indeed, patients with mild dysfunction may benefit from conservative therapies, such as physical therapy and stress management techniques, as described in other research [37,38]. On the other hand, severe dysfunction cases require a multidisciplinary approach, combining dental occlusal adjustments, physiotherapy, and osteopathic interventions, to address both muscular and skeletal dysfunctions.

This holistic approach ensures that treatment is not limited to symptomatic relief but also targets underlying biomechanical dysfunctions, which are often the root cause of the patient’s symptoms. Moreover, the ability to track changes in biomechanical properties over time further enhances the utility of MyotonPRO as a diagnostic and monitoring tool. By providing objective metrics for treatment efficacy, clinicians can refine interventions based on real-time data, a recommendation that is strongly supported [25,26], thus optimizing outcomes and improving the patient’s quality of life.

Finally, we acknowledge that richer statistics, achieved through a larger number of patient samples, could enhance our ability to distinguish and define healthy trends in various populations, specifically regrouped by factors such as age (pediatrics, geriatrics), sex, or environmental conditions. Increasing the sample size will allow us to uncover more nuanced deviations and differences, ultimately leading to more accurate and personalized diagnostic and therapeutic strategies. However, our methodology, which defines a quantitative scale and functionality indices, remains valid and general, and will not need to be changed. This makes it applicable for analysis in different and various biological tissue networks. On the other hand, the lack of a universally accepted gold standard for evaluating TMD presents a considerable challenge, particularly in determining the accuracy of clinical diagnoses. Discrepancies often arise when subjective symptoms reported by patients do not align with objective findings, such as measurements of muscle stiffness or joint mobility. To address this, our approach employs quantitative parameters to enhance diagnostic objectivity compared to traditional methods reliant on self-reported symptoms. This study also faces limitations related to sample size and representativeness, as the recruited subjects represent only a subset of the TMD-affected population [12]. Recognizing these constraints, we have initiated plans to expand and diversify the sample to improve standardization and generalizability of the results.

Future progress in TMD diagnostics will likely benefit from advanced techniques that integrate biomechanical modeling and artificial intelligence tools. These methods offer significant potential to overcome current limitations and refine diagnostic accuracy [31]. Furthermore, integrating multimodal data—including clinical assessments, imaging, and quantitative measurements—into comprehensive diagnostic frameworks is critical. Efforts to establish consensus-based criteria through multicenter collaborations and the adoption of cutting-edge technologies may ultimately lead to a robust and universally accepted gold standard. Despite these limitations, we strongly believe that the application of our methodology over time with a larger number of patients will lead to further advances in understanding functional disorders in several biological tissue networks.

## 5. Conclusions

The integration of biomechanical measurements from the MyotonPRO device into the clinical evaluation of TMDs represents a significant advancement in both diagnostic accuracy and therapeutic planning. Traditional diagnostic methods, which often rely on subjective assessments, may not fully capture the extent of muscle dysfunction, especially in its early stages. The MyotonPRO device provides objective, quantifiable data on key parameters such as muscle stiffness, relaxation time, decrement, and creep, offering a clearer and more comprehensive understanding of masticatory muscle health.

This objective data not only enhance diagnostic precision but also enable the development of personalized, multidimensional treatment plans. By identifying specific biomechanical abnormalities, clinicians can tailor therapeutic interventions more effectively, addressing both the muscular and skeletal components of TMD. A multidisciplinary approach that combines dental, physiotherapeutic, and osteopathic treatments ensures that the root causes of dysfunction are addressed, leading to more sustainable clinical outcomes.

Additionally, the capability to track changes in biomechanical properties over time allows for continuous monitoring of treatment efficacy. This enables clinicians to adjust strategies based on real-time data, helping to prevent the progression of dysfunction and ensuring that patients receive optimal care.

In conclusion, utilizing MyotonPRO alongside traditional diagnostic tools provides a powerful and comprehensive framework for managing TMD. By facilitating earlier detection, more precise treatment, and ongoing monitoring, this integrated approach holds great potential for improving patient outcomes and enhancing the overall management of masticatory muscle dysfunction. The study conducted so far demonstrates that biomechanical and viscoelastic parameters provide valuable insights into understanding TMD. However, we acknowledge that further studies with larger sample sizes are necessary to confirm these findings and improve the clinical applicability of our approach [39]. Furthermore, additional studies are planned to incorporate further evaluation methods and clinical data collection techniques, aiming to enrich our understanding and refine the clinical application of these technologies in diagnosing and treating TMD.

## Data Availability

Data are contained within the article.

## Appendix A

**Questionnaire Structure**

The questionnaire was developed to include validated questions and response scales to ensure the collection of reliable and consistent data. The questions are divided into six main sections:

*1. Frequency and Intensity of Pain:*

Two questions assessing how often the patient has experienced jaw pain in the past six months and the intensity of the pain. Responses are categorized on a scale from “Never” to “Always” and from “Not intense at all” to “Extremely intense” [4].

*2. Impact of Pain on Daily Life:*

Two questions measuring the impact of pain on the patient’s daily activities and ability to work or study. Responses range from “No impact” to “Very severe impact” [40,41].

*3. Sleep Quality:*

Two questions investigating the frequency of sleep difficulties and waking up with jaw pain in the past month, with responses on a scale from “Never” to “Always” [42].

*4. Oral Functionality:*

Two questions evaluating the difficulty in talking for long periods and fully opening the mouth. Responses are classified from “Not difficult at all” to “Extremely difficult” [43].

*5. Psychological Symptoms:*

Two questions exploring the frequency of depressive symptoms in the past month and anxiety in the past week, with responses on a scale from “Never” to “Always” [44,45].

*6. Teeth Clenching/Grinding Frequency:*

Two questions assessing how often the patient clenches or grinds their teeth, and wakes up with clenched or ground teeth. Responses are categorized on a scale from “Never” to “Always”.

**Questionnaire Rating Scale**

For each question, responses were coded on a Likert scale that allows for quantification of TMD symptoms and impact. Responses were classified as follows:

***1. Pain Frequency:***

-*Never*: 0 points

-*Rarely*: 1 point

-*Sometimes*: 2 points

-*Often*: 3 points

-*Always*: 4 points

***2. Intensity of Pain:***

-*Not intense at all*: 0 points

-*Slightly intense*: 1 point

-*Moderately intense*: 2 points

-*Very intense*: 3 points

-*Extremely intense*: 4 points

***3. Impact of Pain on Daily Life (GCPS):***

-*No impact*: 0 points

-*Mild impact*: 1 point

-*Moderate impact*: 2 points

-*Severe impact*: 3 points

-*Very severe impact*: 4 points

***4. Sleep Quality (ISI):***

-*Never*: 0 points

-*Rarely*: 1 point

-*Sometimes*: 2 points

-*Often*: 3 points

-*Always*: 4 points

***5. Oral Functionality (OHIP):***

-*Not difficult at all*: 0 points

-*Slightly difficult*: 1 point

-*Moderately difficult*: 2 points

-*Very difficult*: 3 points

-*Extremely difficult*: 4 points

***6. Psychological Symptoms (PHQ-9, GAD-7, PSS):***

-*Never*: 0 points

-*Rarely*: 1 point

-*Sometimes*: 2 points

-*Often*: 3 points

-*Always*: 4 points

***7. Teeth Clenching/Grinding Frequency:***

-*Never*: 0 points

-*Rarely*: 1 point

-*Sometimes*: 2 points

-*Often*: 3 points

-*Always*: 4 points

**Total Score 0–8:** No dysfunction

- No significant symptoms of TMD. The patient is considered free of temporomandibular disorders.

**Total Score 9–16:** Mild dysfunction

- Mild symptoms of TMD. Pain and impact on daily life are generally manageable with minimal interventions.

**Total Score 17–24:** Moderate dysfunction

- Moderate symptoms of TMD that may interfere with some daily activities. More active treatment is indicated.

**Total Score 25–32:** Severe dysfunction

- Severe symptoms of TMD with a significant impact on quality of life. A multidisciplinary therapeutic approach is required.

**Total Score 33–48:** Extreme dysfunction

- Extremely severe symptoms of TMD with severe functional and psychological impact. Intensive and multidisciplinary management is necessary.
